# Salvage allogeneic hematopoietic stem cell transplantation in a patient with preexisting refractory maxillofacial mucormycosis and severe aplastic anemia: a case report and nursing care model

**DOI:** 10.3389/fmed.2026.1784004

**Published:** 2026-05-05

**Authors:** Wenjuan Cao, Qiuhui Wu

**Affiliations:** Laminar Air-Flow Research Unit, West China Hospital, Sichuan University, Chengdu, China

**Keywords:** allogeneic peripheral blood stem cell transplantation, case report, mucormycosis, nursing care, oral maxillofacial space infection, severe aplastic anemia

## Abstract

**Objectives:**

Patients with severe aplastic anemia (SAA) complicated by mucormycosis face a significantly elevated risk of mortality and present considerable therapeutic challenges. This study aimed to delineate nursing management and propose a structured care model for a case of SAA complicated by mucormycosis undergoing allogeneic hematopoietic stem cell transplantation (HSCT).

**Methods:**

The case involved an 18-years-old female with SAA and a progressive right oromaxillofacial mucormycosis ulcer. During salvage allogeneic HSCT, a nurse-led multidisciplinary team implemented care informed by a structured model. Core interventions were structured around four pillars: (1) a multi-tiered infection control strategy; (2) an innovative wound management protocol combining systemic antifungals with local amphotericin B wet dressings; (3) predictive management of complications; and (4) staged and individualized psychological support. The entire nursing process was guided by the Omaha System as the theoretical framework for problem assessment and intervention.

**Results:**

The patient successfully achieved hematopoietic engraftment. The mucormycosis infection was effectively controlled without further dissemination, and the facial wound decreased significantly in size. A critical Grade 3 cytokine release syndrome was successfully managed. The patient maintained a favorable prognosis at the 1-year follow-up.

**Conclusion:**

This study demonstrates that a structured and nurse-led model of care is pivotal for successfully managing highly complex HSCT patients with life-threatening co-infections. The Omaha System provided a robust and effective framework for delivering systematic and holistic care in this challenging clinical scenario.

## 1 Introduction

Aplastic anemia (AA) is a rare, yet life-threatening disorder of bone marrow failure that is characterized by pancytopenia (1, 2). In China, the reported incidence of AA stands at 0.74 per 100,000. Severe aplastic anemia (SAA), a fulminant subtype of AA, manifests with profound anemia, hemorrhage, and opportunistic infections. Of these effects, invasive fungal infections represent a leading cause of mortality, particularly in patients with prolonged neutropenia (1, 3). Although Oral maxillofacial space infection (OMSI) is a localized anatomical description, it is clinically categorized under rhino-orbital-cerebral (ROC) mucormycosis. According to Kontoyiannis et al. (4) the mortality rate for ROC mucormycosis remains alarmingly high, ranging from 40% to 70%, and frequently exceeds 60% in highly vulnerable populations such as those with hematologic malignancies or bone marrow failure (e.g., aplastic anemia). Current clinical guidelines recommend the immediate initiation of triple immunosuppressive therapy (IST) upon diagnosis, combining two immunosuppressants with a thrombopoietin receptor agonist. For eligible patients, matched sibling donor hematopoietic stem cell transplantation (MSD-HSCT) remains the first-line curative intervention (5–7). However, for patients with refractory SAA or those ineligible for matched sibling donors, salvage allogeneic HSCT from alternative donors represents a potentially curative but high-risk option (7). This risk is exponentially magnified in the presence of an active, invasive fungal infection prior to transplant.

Oral maxillofacial space infection (OMSI) refers to infections within the potential fascial spaces of the maxillofacial region and is clinically characterized by erythema, swelling, localized warmth, pain, and trismus. In the present case, OMSI was complicated by mucormycosis, an aggressive fungal infection characterized by angioinvasion and tissue necrosis that poses significant diagnostic and therapeutic challenges. The management of mucormycosis remains clinically challenging, necessitating early antifungal therapy and aggressive surgical debridement. The pathogenesis of mucormycosis in immunocompromised hosts, such as patients with SAA, involves a confluence of host defense defects and fungal virulence factors. Prolonged and profound neutropenia is the paramount risk factor, as it cripples the primary cellular defense against hyphal invasion (4, 8). Furthermore, underlying immune dysfunction and the use of immunosuppressive therapies impair macrophage and neutrophil phagocytic activity. The fungi of the order Mucorales are angioinvasive, leading to thrombosis, tissue infarction, and necrosis, which facilitates rapid dissemination and hinders antifungal drug penetration (9, 10). Notably, patients with hematological malignancies and those undergoing HSCT are at exceptionally high risk, with mucormycosis carrying mortality rates exceeding 50% in this population, especially with disseminated disease or delayed diagnosis (4, 8, 11). For patients with SAA, the risk is particularly acute. A large retrospective study of infections in AA patients found invasive fungal infections to be a leading cause of mortality (4). Mucormycosis, though less common than aspergillosis or candidiasis, is associated with extreme morbidity due to its destructive nature and mortality rates often exceeding 70%–90% in the setting of persistent neutropenia and hematological malignancy (4, 8, 11, 12). In this context, allogeneic HSCT presents a paradoxical lifeline: it is the only therapy that can definitively correct the underlying bone marrow failure and restore neutrophil production, thereby addressing the root cause of immunodeficiency. Yet, the transplant process itself induces a period of intensified immunosuppression, creating a precarious window where a pre-existing infection like mucormycosis can become fatal (13). Therefore, the decision to proceed with HSCT requires meticulous infection control and a structured supportive care model to navigate this period successfully.

To our knowledge, no published studies have addressed the integrated management of SAA patients complicated by OMSI secondary to mucormycosis. Here, we present a rare case of SAA with refractory maxillofacial mucormycosis who successfully underwent salvage allogeneic HSCT through multidisciplinary collaboration and precision nursing care based on a structured model.

## 2 Case presentation

### 2.1 Patient information

The details of this case are presented in accordance with the CARE reporting checklist^1^. The patient was an 18-years-old female high school senior with no family history of genetic diseases. During her university entrance examination preparation in May 2024, she experienced sudden epistaxis. Laboratory evaluation at an external hospital revealed severe pancytopenia (hemoglobin: 111 g/L; white blood cell count: 0.29 × 10^9^/L; Platelets: 2 × 10^9^/L). Genetic testing for 248 myeloid genes revealed no abnormalities. Bone marrow aspiration confirmed the diagnosis of SAA.

### 2.2 Clinical findings

On admission, the patient presented with pallor and a chronically ill appearance. A 6.5 cm × 4.5 cm full-thickness penetrating ulcer was observed on the right maxillofacial region, covered with gray-black eschar and accompanied by scant yellowish purulent discharge. The ulcer was fixed to the surrounding tissues, causing restricted mouth opening and marked tenderness. Imaging studies [maxillofacial computed tomography (CT)] revealed soft tissue thickening, focal skin discontinuity, facial soft tissue swelling, gas accumulation, and infectious changes. Histopathological examination of the wound tissue revealed broad, ribbon-like, aseptate hyphae with right-angle branching, characteristic of Mucorales. Subsequent next-generation sequencing (NGS) of the wound tissue confirmed the presence of Mucorales, leading to a definitive diagnosis of mucormycosis. The medical diagnosis was acute SAA and right maxillofacial mucormycosis presenting as a large ulcer.

### 2.3 Timeline

**FIGURE 1:**
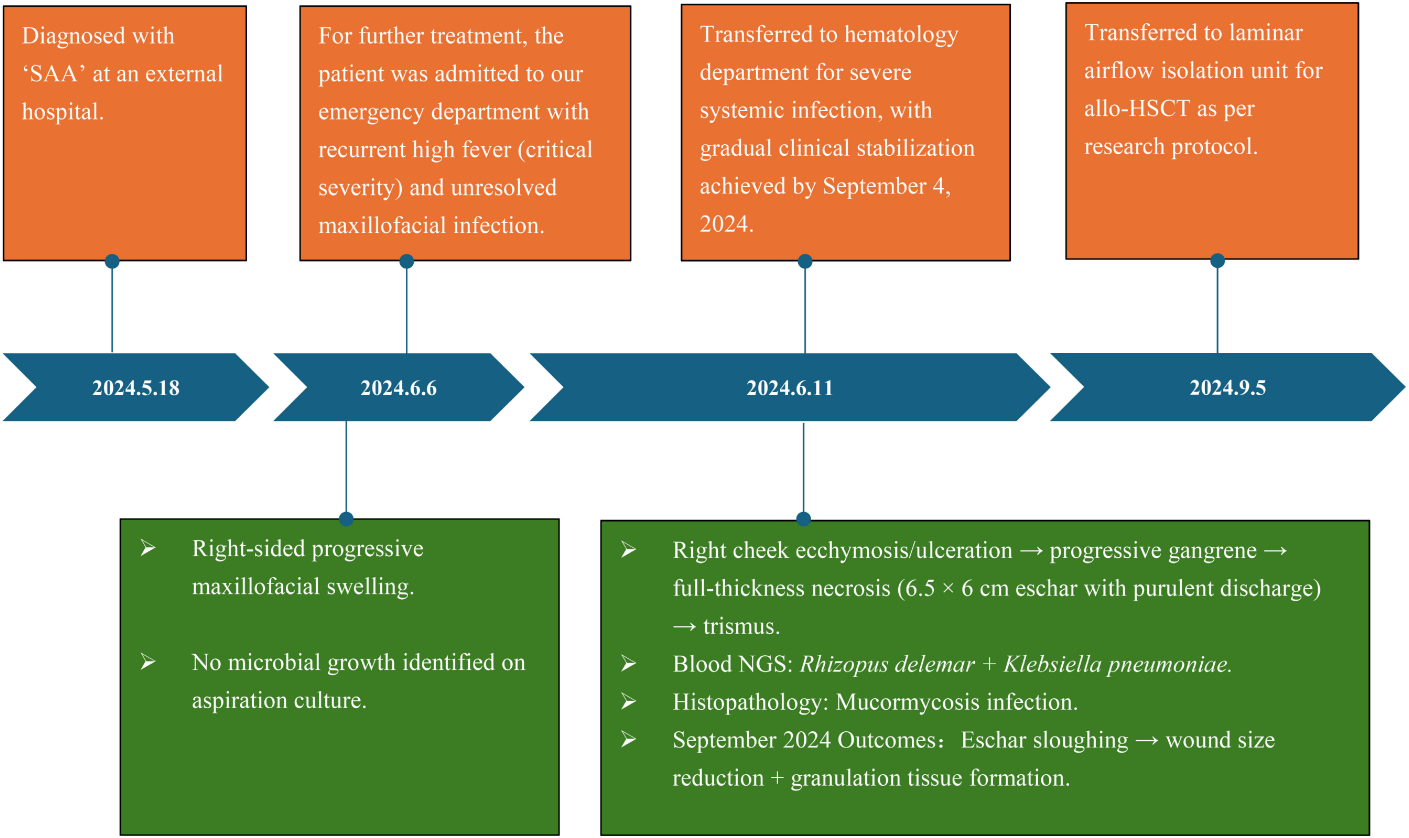
The treatment timeline for an 18-years-old female patient with SAA combined with OMSI (mucormycosis). This timeline illustrates the key diagnostic, therapeutic, and nursing milestones from initial diagnosis at an external hospital to preparation for allo-HSCT. The timeline highlights the rapid progression of fungal infection during a period of severe neutropenia and the subsequent stabilization of the patient’s condition following aggressive anti-infective and supportive care. Key microbiological findings are included (e.g., *Rhizopus delemar* and *Klebsiella pneumoniae* detected by the next-generation sequencing of blood). The eventual reduction in wound size and the formation of granulation tissue by September 2024 created the necessary conditions for salvage transplantation. SAA, severe aplastic anemia; OMSI, oral maxillofacial space infection; allo-HSCT, allogeneic hematopoietic stem cell transplantation; NGS, next-generation sequencing.

**FIGURE 2:**
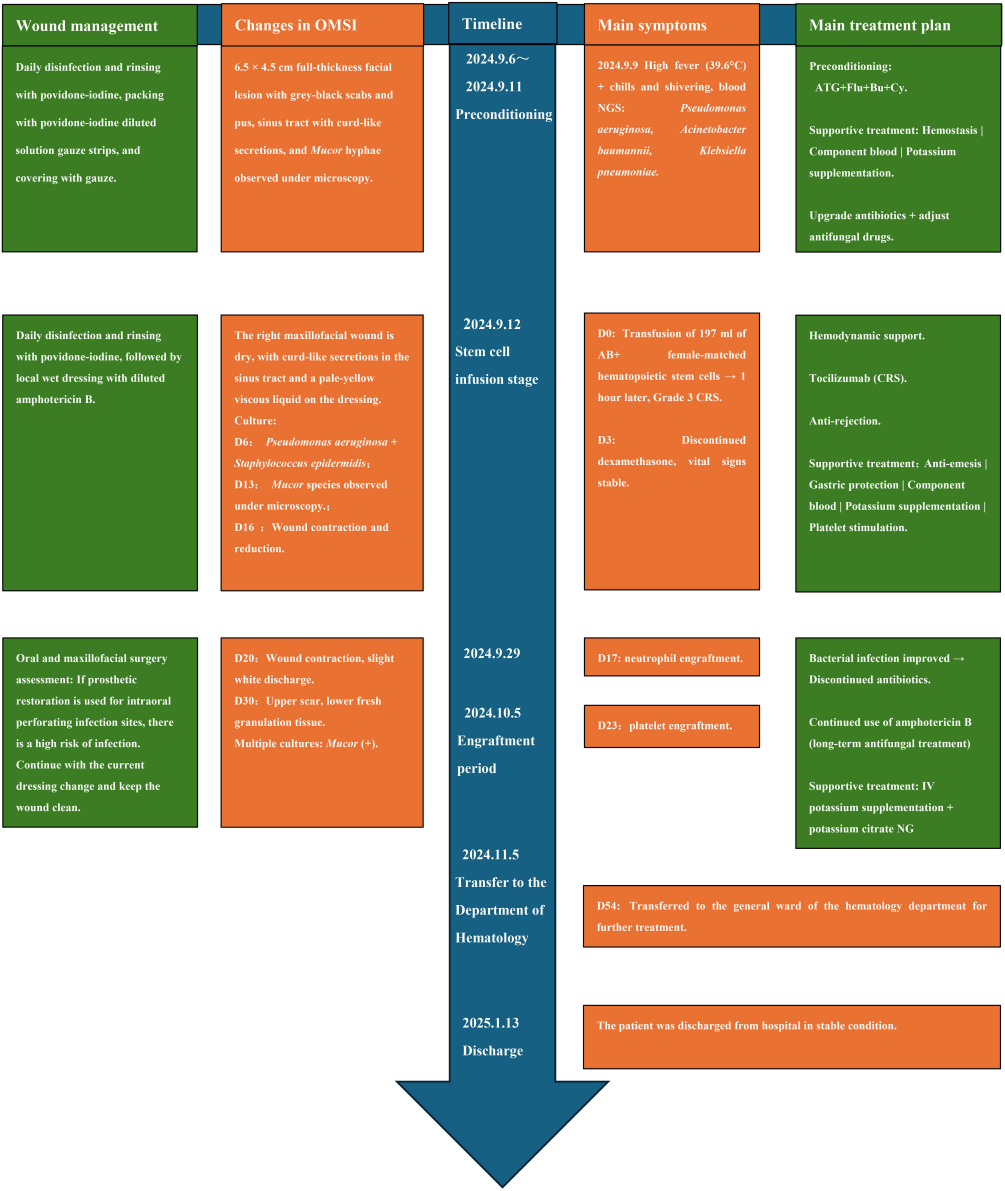
Timeline for patient transfer to the laminar flow ward for allo-HSCT. This figure outlines the key medical and nursing interventions, microbiological findings, and clinical responses from the initiation of the preconditioning regimen through to neutrophil and platelet engraftment, and eventual discharge. Key events include the management of bacterial co-infections and CRS, as well as the evolution of the mucormycosis wound under a structured local and systemic antifungal protocol. allo-HSCT, allogeneic hematopoietic stem cell transplantation; CRS, cytokine release syndrome; NGS, next-generation sequencing; ATG, anti-thymocyte globulin; Flu, fludarabine; Bu, busulfan; Cy, cyclophosphamide. IV, intravenous; NG, nasogastric.

The patient’s disease progression is illustrated in Figure 1 and Supplementary Figure 1. Prolonged severe neutropenia led to rapid progression of the maxillofacial fungal infection, with worsening tissue necrosis and expansion into a full-thickness penetrating ulcer. The condition stabilized after 2 months of anti-infective and supportive treatment. On the 5th September 2024, the patient was transferred to our department for salvage allo-HSCT; the detailed treatment process is shown in Figure 2. The patient’s clinical course and antifungal treatment regimen are presented in Table 1.

**TABLE 1:** Integrated pharmacological timeline: antifungal and antibacterial management from induction to outpatient follow-up.

Phase	Time	Antifungal therapy	Antibacterial therapy	Adjustment/rationale
Introduction (anti-infective therapy) 2024.6.11 ∼ 2024.9.5	6.11	Imipenem/cilastatin: 500 mg, q8h, IV; Norvancomycin: 800 mg, q12h, IV.	Isavuconazonium: 200 mg, qd, IV; Voriconazole tablets: 200 mg, q12h, PO.	Empirical therapy: sepsis, recurrent fever, WBC 0.64 × 10^9^/L, bruising and ulceration of the maxillofacial skin.
	6.19	Linezolid and glucose injection: 600 mg, q12h, IV. Ceftazidime-avibactam: 2.5 g, q8h, IV; Compound sulfamethoxazole tablets: 0.8 g, qd, PO.	Esaconazole: 200 mg, qd, IV; Voriconazole tablets: 200 mg, q12h, PO.	Targeted therapy: the sputum culture yielded *Stenotrophomonas maltophilia*, and the ulceration area of the maxillofacial region has expanded.
	7.2	Imipenem/cilastatin: 500 mg, q8h, IV; Linezolid and glucose injection: 600 mg, q12h, IV. Compound sulfamethoxazole tablets: 0.8 g, qd, PO.	Amphotericin B deoxycholate (AmBd): 25 mg, qd, IV; Esaconazole: 200 mg, qd, IV.	Targeted therapy: Blood NGS: *Rhizopus delemar*, *Klebsiella pneumoniae*. Respiratory pathogen PCR: methicillin-resistant *Staphylococcus* positive (MRSA).
Preconditioning 2024.9.6 ∼ 2024.9.11	9.9	Linezolid and glucose injection: 600 mg, q12h, IV. Ceftazidime-avibactam: 2.5 g, q8h, IV; Compound sulfamethoxazole tablets: 0.8 g, qd, PO.	Liposomal amphotericin B (L-AmB): 200 mg, qd, IV; Esaconazole: 200 mg, qd, IV; Local AmBd 5 mg wound dressing, qd.	Targeted therapy: High fever (39.6 °C), Blood NGS: *Pseudomonas aeruginosa*, *Acinetobacter baumannii*, *Klebsiella pneumoniae*; Facial lesion fungal stain: Mucorales hyphae positive; Targeted local therapy to control maxillofacial foci and limit systemic exposure/toxicity.
Stem cell infusion stage 2024.9.12	9.12	Cefoperazone/sulbactam: 3 g, q8h, IV; Compound sulfamethoxazole tablets: 0.8 g.	Liposomal amphotericin B (L-AmB): 150 mg, qd, IV; Esaconazole: 200 mg, qd, IV; Local AmBd 5 mg wound dressing, qd.	Targeted therapy: post-CRS intervention: normotensive and afebrile. Empirical therapy: clinically stable; de-escalated to low-dose maintenance bridging.
	9.17	Piperacillin/tazobactam: 4.5 g, q8h, IV; Compound sulfamethoxazole tablets: 0.8 g.		
Engraftment period 2024.9.29 ∼ 2024.11.4	9.29	Ceftazidime: 1 g, q8h, IV; Compound sulfamethoxazole tablets: 0.8 g.		Targeted therapy: neutrophil engraftment. Eight serial maxillofacial secretion cultures: all positive for *Pseudomonas aeruginosa*; fungal fluorescence staining revealed fungal hyphae suggestive of Mucorales.
Maintenance period 2024.11.5 ∼ 2025.1.13	11.20	Antibiotics discontinued.	Liposomal amphotericin B (L-AmB): 100 mg → 50 mg, qd, IV; Esaconazole capsules: 200 mg, qd, PO; Local AmBd 5 mg wound dressing, qd.	Empirical therapy: The dosage reduction of antimicrobial agents was an empirical de-escalation based on clinical stability and the preservation of renal function.
Outpatient follow-up	5.1 ∼ Present	/	Esaconazole capsules: 200 mg, qd, PO.	Transitioned to oral monotherapy for long-term maintenance.

qd, once daily; q8h, every 8 hours; q12h, every 12 hours; biw, twice a week; IV, intravenous; PO, by mouth (oral); Ambd, amphotericin B deoxycholate; L-AmB, liposomal amphotericin B.

### 2.4 allo-HSCT and immune reconstitution

In September 2024, the patient received a haploidentical allo-HSCT from her mother (HLA 6/12 matched, ABO-mismatched: AB+ to B+). A myeloablative conditioning regimen (ATG + Flu + Bu + Cy) was administered from day −7 to −2. On Day 0, 197 ml of stem cells were infused. The immediate post-infusion period was complicated by Grade 3 CRS (fever and hypotension), which resolved promptly with Tocilizumab. Successful hematopoietic reconstitution was achieved, with neutrophil and platelet engraftment confirmed on Day + 17 and Day + 23, respectively. This immune recovery was pivotal for controlling the localized mucormycosis (Figure 2).

### 2.5 Nursing assessment

A comprehensive nursing assessment was conducted using the Omaha System problem classification scheme to systematically identify and categorize the patient’s health problems (14). This framework, encompassing the Environmental, Psychosocial, Physiological, and Health-related behaviors domains, ensured a holistic understanding of her complex needs. This framework, encompassing the Environmental, Psychosocial, Physiological, and Health-related behaviors domains, ensured a holistic understanding of her complex needs. The following four priority problems were identified: (1) balancing infection control and immunosuppression; (2) care for refractory fungal infections; (3) long-term hospitalization psychological intervention and (4) proactive management of transplantation complications.

### 2.6 Nursing measures

#### 2.6.1 Multidisciplinary collaboration and anticipatory care

A nurse-led multidisciplinary team (MDT) was established, integrating specialists from transplantation, maxillofacial surgery, infectious diseases, and clinical nutrition. This team developed precision protocols for antifungal wound management, CRS prevention, and nutritional support. The nursing team implemented early-warning and response systems for potential complications including Grade ≥ 3 CRS, septic shock, and hypovolemic shock, enabling rapid intervention and contributing significantly to the successful outcome.

#### 2.6.2 Infection control throughout the transplant process

Given the patient’s pre-existing mucormycosis and profound immunosuppression risk, a rigorous three-tiered strategy was deployed to prevent secondary infections (1, 3).

(1) Environmental management: the patient was placed in protective isolation within a laminar airflow (Class 100) single room, with strict enforcement of hand hygiene and standard precautions.
(2) Barrier protection: measures included daily disinfection of personal utensils, provision of sterile clothing, and intensified oral and perianal care to reinforce mucosal and skin integrity.
(3) Close monitoring of changes in symptoms and signs, along with timely microbial monitoring. Prompt microbial investigation, including blood culture and NGS, identified *Pseudomonas aeruginosa* and other bacteria in the blood, and *Mucor* mycelium in the facial wound. Given the identification of a member of the Mucorales order and in accordance with first-line treatment guidelines for mucormycosis (8), therapy was immediately escalated to liposomal amphotericin B and combined antibiotics (ceftazidime/avibactam and linezolid). Subsequently, the body temperature gradually returned to normal. In this case, linezolid was administered to cover Methicillin-resistant *Staphylococcus aureus* (MRSA). As detailed in Table 1, the antifungal regimen was phased according to the patient’s clinical status and renal function. Systemic therapy was de-escalated from a combination of L-AmB and isavuconazole to oral isavuconazole monotherapy only after successful neutrophil engraftment and documentation of wound stabilization.

#### 2.6.3 Mucormycosis wound protocol

Currently, domestic and international guidelines, along with expert consensus on the diagnosis and treatment of cutaneous mucormycosis (8), recommend systemic antifungal therapy combined with surgical treatment. For refractory mucormycosis infections, the local application or injection of amphotericin B may also be considered (15).

An innovative “systemic treatment + local management” model was adopted for our patient, which was applied in five steps. (1) Systemic antifungal therapy with intravenous liposomal amphotericin B and oral itraconazole, with concurrent potassium supplementation to prevent hypokalemia; (2) Local wound care using amphotericin B (2 mg/ml) wet dressings; (3) Comprehensive supportive measures including individualized enteral nutrition, enhanced oral care with Rehabilitation New Solution and nystatin mouthwash, and adequate pain management; (4) Regular monitoring and documentation of wound characteristics; and (5) Strict isolation precautions during all procedures. After 61 days of intervention, the maxillofacial mucormycosis infection did not spread and gradually reduced in size, creating appropriate conditions for subsequent treatment.

The cornerstone of our antifungal strategy was the synergistic combination of systemic and local therapy. While L-AmB provided systemic coverage, the angioinvasive nature of the *Mucor* mycelium often results in localized thrombosis and necrotic barriers that impede systemic drug delivery. Therefore, local AmBd (5 mg) wound dressings were applied daily to the maxillofacial lesion. This adjunctive measure aimed to deliver a high concentration of the fungicidal agent directly to the necrotic tissue, a strategy supported by recent clinical evidence for refractory mucormycosis.

#### 2.6.4 Predictive management of cytokine release syndrome

Cytokine release syndrome (CRS), particularly Grade 3 or higher, represents a critical complication following haploidentical HSCT and can rapidly progress to shock and multi-organ failure (16). In haploidentical transplants, the incidence of severe CRS reaches 12% (17). Our patient developed Grade 3 CRS 1 h post-transplantation, presenting with high fever (39.3 °C), hypotension (79/37 mmHg), and elevated IL-6 (410.5 pg/mL). The multidisciplinary team immediately implemented a four-step emergency protocol: (1) hemodynamic support with norepinephrine and fluid resuscitation; (2) immunomodulation using tocilizumab and dexamethasone; (3) intensive monitoring of vital signs, inflammatory markers, and fluid balance; (4) organ function protection. Through this structured approach, the patient’s vital signs normalized within 3 days, highlighting the efficacy of proactive CRS management.

#### 2.6.5 Psychological care during stem cell transplantation

Our patient was a female high school senior in her teenage years with an introverted personality. Due to prolonged isolation treatment and destructive lesions in the maxillofacial region, she was experiencing complex emotions such as self-image disturbance, anxiety, and fear. In addition, the transplantation process involves complexity, uncertain outcomes, and high risks of rejection, placing significant stress and psychological distress on the patient’s caregivers (18). These effects may exert adverse effects on a patient’s long-term treatment and quality of life. Our nursing team implemented personalized psychological interventions based on the characteristics of the three key stages of HSCT, as follows:

(1) Stage-Matched Support: tailored psychological strategies were delivered across transplant phases: the pretreatment phase focused on building therapeutic alliance and managing treatment-related symptoms; the reinfusion phase emphasized anticipatory guidance and peer support; and the engraftment phase promoted self-management and future surgical planning.
(2) Structured Initiatives: key activities included a weekly “Family Communication Day” for education and counseling, continuous support via a WeChat-based care platform, and participation in the “Sunshine Angel” psychological support program.
(3) Therapeutic Communication: the care team consistently employed enhanced communication techniques, including active listening, non-verbal reassurance, and meaning-centered approaches, to foster resilience and facilitate a transition from passive acceptance to active coping.

This comprehensive approach effectively supported the patient’s psychological adaptation throughout her treatment journey (18). Objective evaluations substantiated these clinical observations: the Huaxi Emotional Index score decreased from 3 at admission to 0 prior to discharge, while the Pittsburgh Sleep Quality Index score adjusted from 2 to 1, indicating maintained emotional stability and sleep quality.

#### 2.6.6 Follow-up and outcomes

**FIGURE 3:**
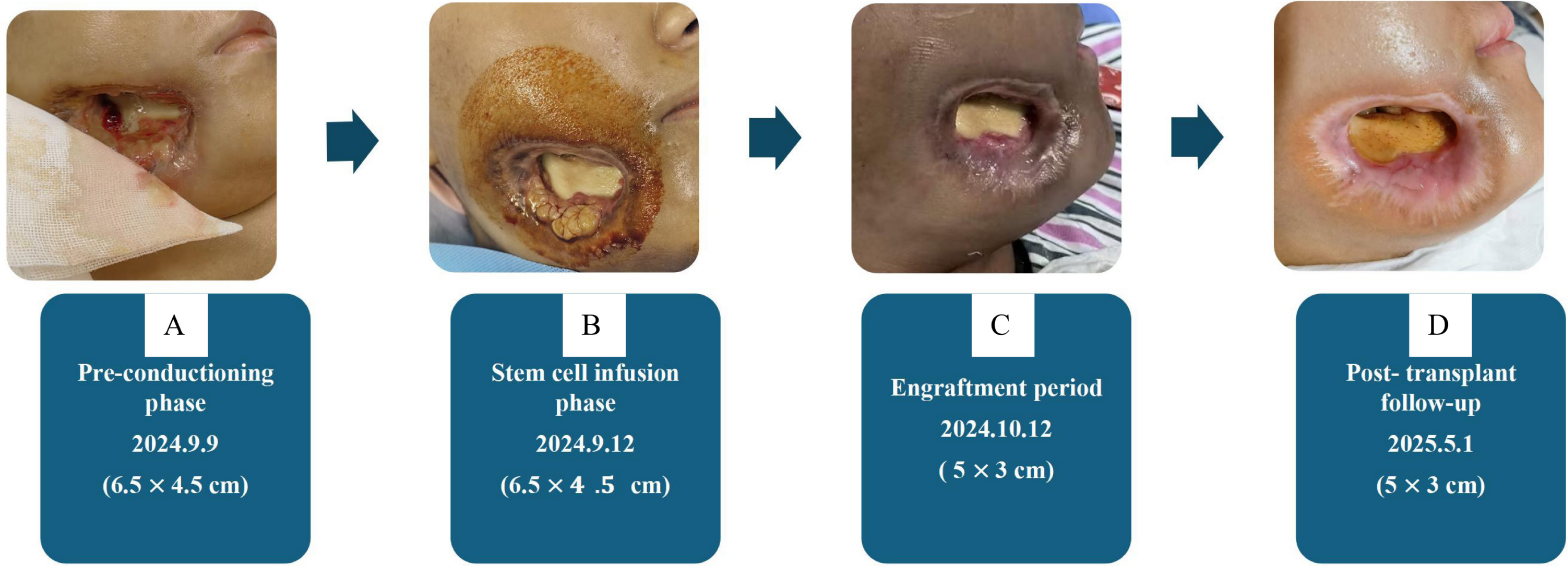
Changes in the infected wound in the right oral maxillofacial space. Serial clinical photographs demonstrate the progressive reduction of the right oromaxillofacial mucormycosis wound during various phases of HSCT, under a regimen of combined antifungal therapy and nursing care. **(A)** Initial presentation showing the extensive necrotic ulcer with eschar; **(B–D)** sequential follow-up images showing progressive reduction in wound size, formation of healthy granulation tissue, and contraction of the surrounding skin margins. Published with the patient’s written informed consent. HSCT, hematopoietic stem cell transplantation.

The patient successfully achieved hematopoietic engraftment. The mucormycosis infection was controlled, and the facial wound decreased significantly, as shown in Figure 3. The patient’s post-transplant recovery was characterized by a gradual normalization of the immune system. Although secondary maxillofacial reconstruction was originally scheduled for late 2025, but the procedure was postponed to prioritize sustained hematopoietic stability. At the most recent follow-up in April 2026, the patient showed complete hemogram recovery and remains free of fungal recurrence. Consequently, the maxillofacial flap repair is now scheduled for the near future.

## 3 Discussion

While contemporary therapies including allogeneic HSCT have significantly improved long-term survival for patients with SAA, achieving 5-years survival rates of 80%–90% (6), the presence of an active, invasive fungal infection like mucormycosis drastically elevates the risk of transplant failure and mortality (8). This peril is magnified when the infection involves the oral and maxillofacial region–an area with complex anatomy where infections can rapidly disseminate, leading to catastrophic outcomes such as sepsis and airway compromise.

Given this strategic paradox, a MDT, comprising hematologists, infectious disease specialists, and surgeons, conducted a rigorous risk-benefit assessment. The decision to proceed with salvage allogeneic HSCT was predicated on the understanding that durable immune reconstitution–primarily through the recovery of functional neutrophils and cellular immunity–represents the cornerstone for the definitive clearance of invasive mucormycosis. We determined that the potential for long-term survival afforded by successful hematopoietic reconstruction outweighed the near-certain fatality associated with persistent pancytopenia, a condition that renders invasive fungal infections recalcitrant to even the most intensive pharmacological interventions. To mitigate risks, a strategic window of opportunity was identified following 2 months of aggressive pre-transplant therapy, which had successfully stabilized and localized the maxillofacial lesion. This ensured the procedure was performed during a period of relative clinical stability, with a structured, nurse-led care model providing an essential safeguard to navigate the complex peri-transplant challenges.

**FIGURE 4:**
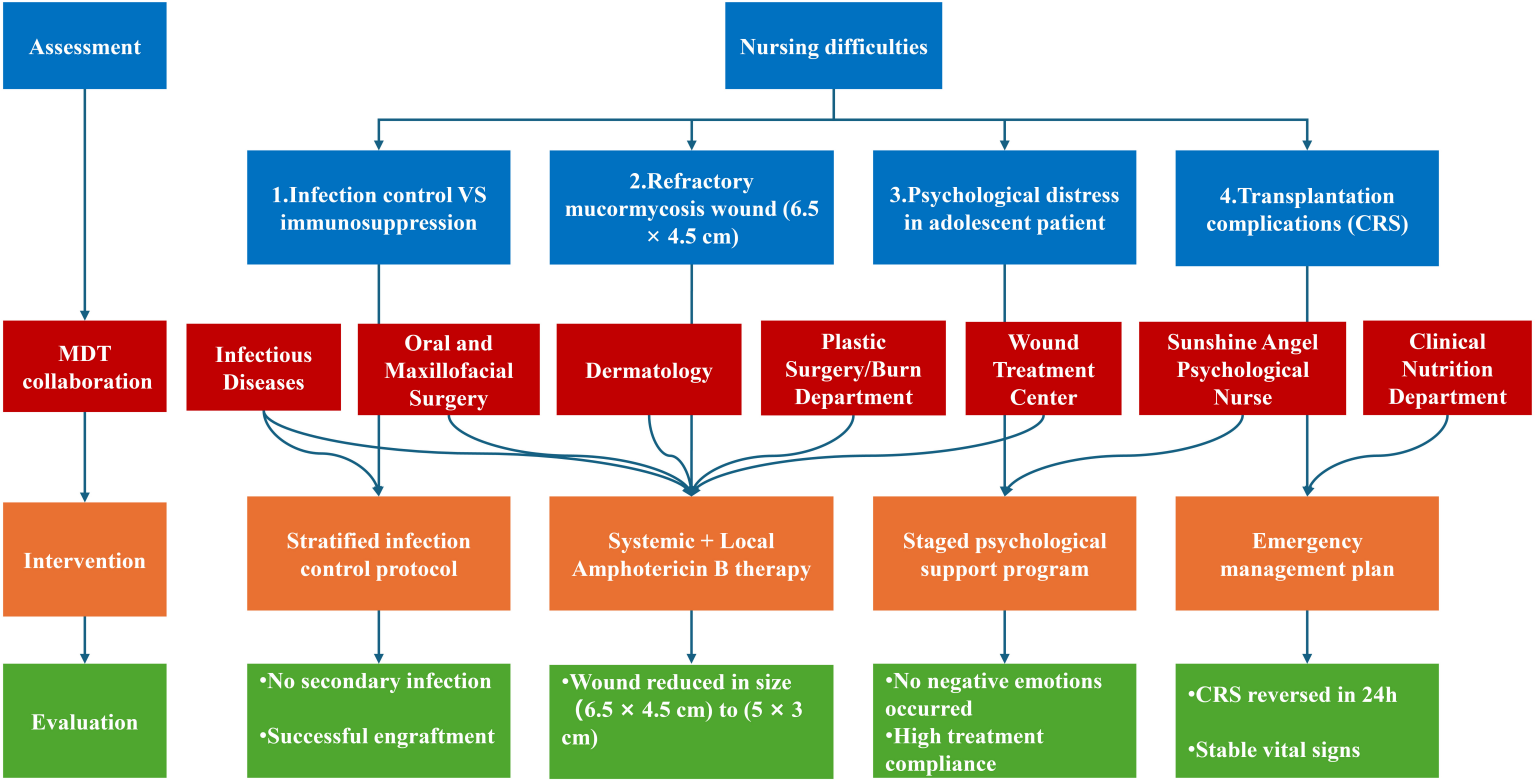
Nursing management pathway supported by a multidisciplinary collaboration for HSCT with mucormycosis. This flowchart illustrates the core nursing process, from assessment of initial difficulties through implementation of targeted interventions to evaluation of outcomes. Dashed lines indicate specific contributions from MDT specialists to each nursing protocol, highlighting the collaborative care model that achieved successful results in this complex case. HSCT, hematopoietic stem cell transplantation; MDT, multidisciplinary team; CRS, cytokine release syndrome.

As visually summarized in Figure 4, the successful outcomes were achieved through a coordinated approach underpinned by the Omaha System (14), with key successes including:

First, we established a model, which broke down professional barriers and improved efficiency; Secondly, the innovative wound protocol combining systemic and local amphotericin B, which effectively controlled the refractory infection; Thirdly, a proactive nursing system was established, which utilized consensus-guided CRS protocols and shifted from reactive response to prevention; At last, comprehensive, phased psychosocial support was tailored to an adolescent patient, which improved adherence and quality of life.

The Omaha System served as the foundational framework that structured this entire care process. Its problem classification scheme enabled a holistic and systematic assessment, moving beyond the immediate physiological crisis to identify interrelated problems in the Psychosocial (e.g., self-image disturbance, anxiety) and Health-related behaviors (e.g., coping with long-term isolation) domains. This comprehensive problem list directly informed the development of our four-pillar intervention model. Furthermore, the Omaha System’s intervention scheme provided a standardized language for documenting care, ensuring consistency and continuity across shifts and disciplines. Its problem rating scale for outcomes offered a method to quantitatively evaluate progress on issues like wound healing and psychological adaptation, shifting care from intuitive to evidence-based. In this case, the System’s efficacy was demonstrated by its role in translating an overwhelming clinical scenario into a manageable series of prioritized, actionable problems, thereby coordinating the multidisciplinary team’s efforts toward unified, patient-centered goals.

The limitations of this study lie in the need for further refinement of standardized protocols for local antifungal therapy. Future research should investigate optimal parameters for the administration of amphotericin B (e.g., concentration and duration), and the development of novel drug delivery formulations (e.g., sustained-release gels) to ensure the safety and efficacy of local therapy.

## 4 Conclusion

This case provides an important reference for the treatment and care of complex hematological diseases complicated by fungal infections in special locations. The innovative practices developed during our patient’s clinical management have significant clinical value for improving the success rate of treatment for similar cases.

## Data Availability

The original contributions presented in this study are included in the article/Supplementary material, further inquiries can be directed to the corresponding author.

## Footnotes

1
https://www.care-statement.org/downloads/

## Supplementary material

Supplementary Figure 1 Timeline of serial radiological surveillance confirming localized maxillofacial mucormycosis. These representative CT images demonstrate the localized nature of the infection throughout the therapeutic process. The serial scans of the chest and abdomen show no evidence of pulmonary or visceral fungal dissemination, confirming that the mucormycosis remained strictly confined to the right maxillofacial region. These radiological findings provided the essential clinical evidence to support the decision for high-risk hematopoietic stem cell transplantation and illustrate the effectiveness of the infection control strategy.
