# Supplementary figures and images for: Salvage allogeneic hematopoietic stem cell transplantation in a patient with preexisting refractory maxillofacial mucormycosis and severe aplastic anemia: a case report and nursing care model
Source: Front Med (Lausanne). 2026 May 5;13:1784004. doi: 10.3389/fmed.2026.1784004 (PMC13183610; doi:10.3389/fmed.2026.1784004)

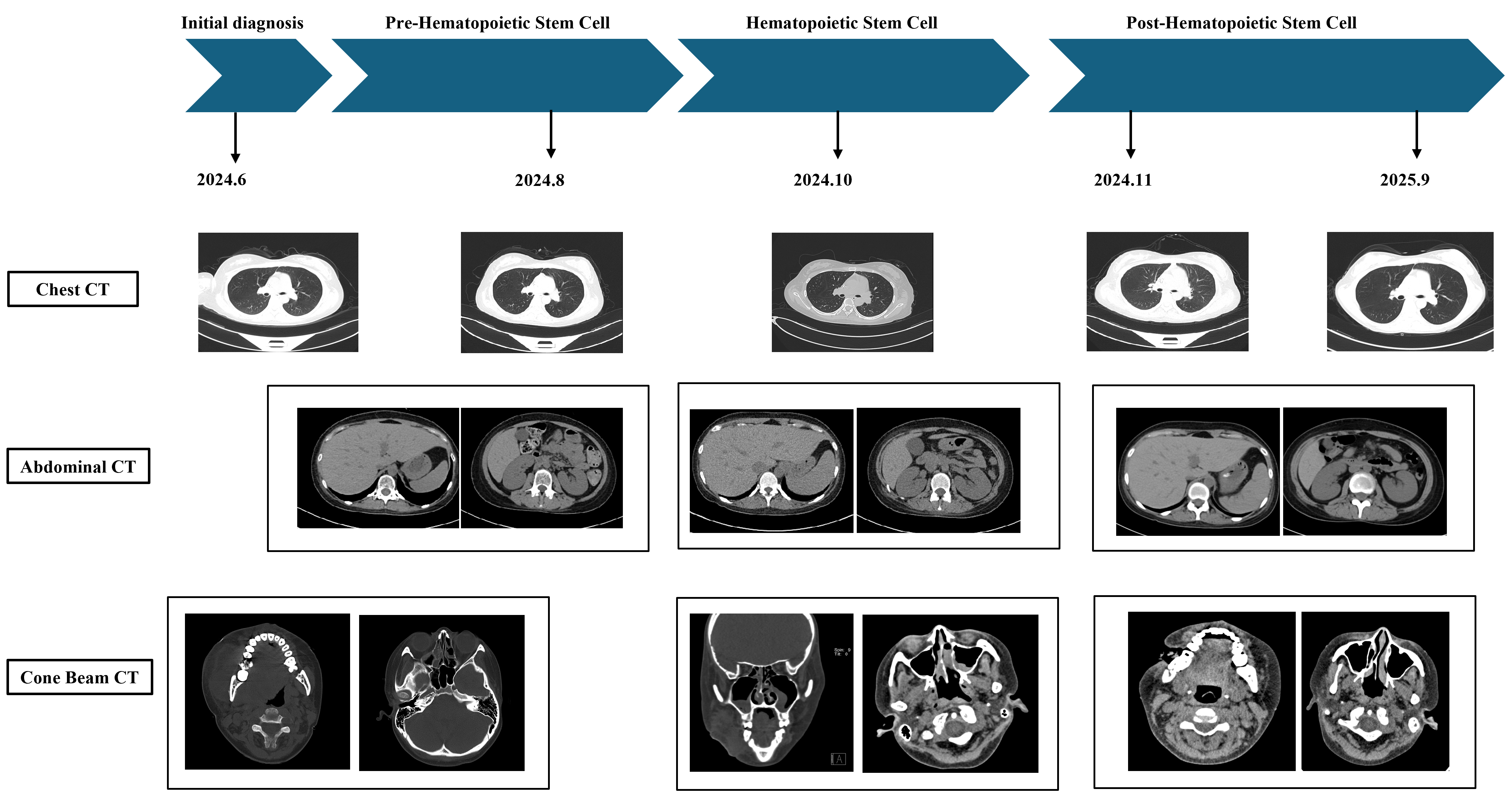

Supplement: Supplementary Figure 1 — Timeline of serial radiological surveillance confirming localized maxillofacial mucormycosis. These representative CT images demonstrate the localized nature of the infection throughout the therapeutic process. The serial scans of the chest and abdomen show no evidence of pulmonary or visceral fungal dissemination, confirming that the mucormycosis remained strictly confined to the right maxillofacial region. These radiological findings provided the essential clinical evidence to support the decision for high-risk hematopoietic stem cell transplantation and illustrate the effectiveness of the infection control strategy. [file Image_1.tif]
